# Non-invasive tracking of disease progression in young dystrophic muscles using multi-parametric MRI at 14T

**DOI:** 10.1371/journal.pone.0206323

**Published:** 2018-10-26

**Authors:** Joshua S. Park, Ravneet Vohra, Thomas Klussmann, Niclas E. Bengtsson, Jeffrey S. Chamberlain, Donghoon Lee

**Affiliations:** 1 Department of Radiology, University of Washington, Seattle, WA, United States of America; 2 Department of Neurology, University of Washington, Seattle, WA, United States of America; 3 Senator Paul D. Wellstone Muscular Dystrophy Cooperative Research Center, University of Washington, Seattle, WA, United States of America; 4 Department of Biochemistry, University of Washington, Seattle, WA, United States of America; 5 Department of Medicine, University of Washington, Seattle, WA, United States of America; University of Sydney, AUSTRALIA

## Abstract

In this study, multi-parametric magnetic resonance imaging (MRI) was conducted to monitor skeletal muscle changes in dystrophic (*mdx*^*4cv*^) and age-matched control (*C57BL/6J*) mice starting at 3 weeks of age. The objective of this study was to evaluate and characterize changes in muscle tissue characteristics of hind limbs in young, dystrophic mice using MRI. *Mdx*^*4cv*^ (n = 25) and age-matched *C57BL/6J* (n = 5) were imaged at 3, 5, 7, 9, and 11 weeks of age. Multiple MR measurements were taken from the tibialis anterior, gastrocnemius, and soleus muscles. There were significant differences between dystrophic and control groups for all three muscle types when comparing transverse relaxation times (T_2_) in lower hind limb muscles. Additionally, fractional anisotropy, radial diffusivity, and eigenvalue analysis of diffusion tensor imaging also demonstrated significant differences between groups. Longitudinal relaxation times (T_1_) displayed no significant differences between groups. The earliest time points in the magnetization transfer ratio measurements displayed a significant difference. Histological analysis revealed significant differences in the tibialis anterior and gastrocnemius muscles between groups with the *mdx* mice displaying greater variability in muscle fiber size in later time points. The multi-parametric MRI approach offers a promising alternative for future development of a noninvasive avenue for tracking both disease progression and treatment response.

## Introduction

The muscular dystrophies are a group of inherited diseases that are characterized by progressive muscle wasting and weakness. Duchenne muscular dystrophy (DMD) is the most prevalent, and severe, form of muscular dystrophy affecting approximately 1 in every 5000 male births [1, 2]. DMD is an X-linked recessive degenerative condition with no cure and an average life expectancy of approximately 25–30 years [3]. Mutations in the dystrophin gene at locus Xp21 result in abnormal or absent expression of dystrophin: a 427 KDa cytoskeletal protein [4]. Dystrophin is responsible for linking actin filaments underlying the muscle sarcolemma to the extracellular matrix via assembly of the dystrophin-glycoprotein complex (DGC). Disruption of the DGC reduces lateral transmission of forces from muscle cells, affecting membrane integrity and intracellular signaling, which leads to necrosis and replacement of muscle with fatty and connective tissues [5–7]. This steady and progressive muscle deterioration ultimately results in respiratory and cardiac failure.

The most commonly used animal models in preclinical studies of DMD are various strains of *mdx* mice. Previous studies have shown critical periods of muscle degeneration and regeneration within the first 2–4 months of life, peaking between weeks 4 and 5 [8]. Additionally, differences and abnormalities in muscular integrity have been seen in cardiac muscle as early as one month of age while skeletal muscles have shown differences for mice as early as 5 weeks of age [9]. After this early period, necrosis gradually decreases until a low and persistent level is reached in the adult *mdx* mouse [10]. The phenotype observed in the *mdx* mouse is much less severe than in human DMD patients. Regardless, there are similar aspects between the *mdx* and DMD phenotype such as centrally nucleated muscle fibers, widespread myofiber necrosis, variations in myofiber size, and an increased susceptibility to contraction-induced injury [6, 10]. One of the significant hurdles encountered so far is lack of sensitive quantitative biomarkers to monitor disease progression in both preclinical and clinical models of DMD. Both preclinical and clinical assessment of tissue characteristics has historically been achieved through surgical biopsy that fails to provide detailed information throughout the whole muscle because the invasive nature of the procedure greatly restricts both the sampling regions and sampling frequency [11–14].

Over the last decade, the emergence of non-invasive measures has provided an alternative means for acquiring such tissue information without the same limitations [9, 15, 16]. Indeed, magnetic resonance imaging (MRI) has been used to monitor disease progression in both human and preclinical models. One of the limitations of the aforementioned studies is that a single MRI parameter was used to monitor the disease progression in preclinical and human populations [17–21]. However, recent utilization of multi-parametric MRI (mp-MRI) involving a host of different parameters; such as longitudinal (T_1_) and transverse (T_2_) relaxation times, magnetization transfer ratio (MTR), and diffusion MRI, have been incorporated to study treatment effects of adeno-associated viral (AAV) vector-mediated gene therapy in *mdx* mice [22, 23]. These multimodal MR approaches to tissue characterization have shown the ability to detect pathological changes in a variety of diseases at the cellular level.

The previously documented cyclical pattern of degeneration, regeneration, and inflammation; which stabilizes around 11–12 weeks of age, is poorly characterized beyond the gold standard MR measures of T_1_ and T_2_. Because of this, there remains an incomplete understanding of the cellular processes occurring during this critical time of development and how to utilize MR to capture this data. This period of cyclical changes has been observed but not characterized for possible treatment at such an early age using many MR parameters [24]. A more complete understanding of the disease progression is necessary in order to open possible avenues for future treatment regimens and translation into human clinical studies.

The goal of this study was to evaluate and characterize changes in muscle tissue characteristics in young *mdx*^*4cv*^ mice using *in vivo* MRI and histology to better understand the progression of the disease at this early stage to enable development of potential therapeutic plans exploring the possibility of better treatment outcomes.

## Materials and methods

### Animals

In this study, we conducted multi-parametric MRI for two groups of mice: one group of normal (*C57BL/6J*) mice and one group of *mdx*^*4cv*^ (*B6Ros*.*Cg-Dmd*^*mdx-4Cv*^*/J*) mice [25]. All mice were housed and treated in strict accordance with the National Institutes of Health (NIH) Guide for the Care and Use of Experimental Animals and approvals from the Institutional Animal Care and Use Committee (IACUC, protocol number 4210–01) of the University of Washington. The mice were housed in specific pathogen free (SPF) facilities running 12:12 light/dark cycles at ambient temperatures of 22–23°C with access to food and water ad libitum. All of these conditions were maintained for the duration of the study.

### Study design

Both *mdx*^*4cv*^ and *C57BL/6J* mice were obtained at 3 weeks of age. These groups were longitudinally tracked beginning at 3 weeks of age and ending at 11 weeks of age. The mice were imaged utilizing T_1_, T_2_, diffusion weighted imaging (DWI), diffusion tensor imaging (DTI), and magnetization transfer imaging (MTI) every two weeks for a total of 5 time points to monitor disease progression and differences between the groups. Additional groups of 5 *mdx*^*4cv*^ mice were imaged at each time point and subsequently sacrificed and used for histological assessments. In total, there were *mdx*^*4cv*^ (n = 25) mice along with the age matched normal *C57BL/6J* (n = 5) mice that were imaged at 3, 5, 7, 9, and 11 weeks of age as part of the longitudinal or single time point groups. For histological measurements, mice were anesthetized and euthanized by cervical dislocation while under anesthesia. All mice were euthanized following the conclusion of the study.

### MR data acquisition

The mice were imaged on a Bruker 14T Avance 600 MHz/89 mm wide-bore vertical MR spectrometer (Bruker Corp., Billerica, MA). While being imaged, mice were under isoflurane anesthesia (1.5–2%) and kept from prolonged imaging sessions to minimize animal stress. Once under anesthesia, the mice were secured by a custom-built mouse holder. The mice were monitored for respiratory rate throughout the duration of the imaging time with the animal’s ambient temperature kept at 30°C. The high resolution MRI protocol included scout imaging (gradient echo; TR (repetition time)/TE (echo time) = 100/3.42 ms) and planning for image planes (multi-slice RARE (rapid acquisition with refocused echoes): TR/TE = 667.54/4.47 ms). **T**_**1**_ **measurements**: Multi-slice, fat suppressed images with refocused echoes (TR/TE = 5500, 3000, 1500, 1000, 385.8/9.66 ms), matrix size = 256 x 128, FOV 25.6 x 25.6 mm were used for T1 measurements. **T**_**2**_ **measurements**: The quantitative T_2_ measurements utilized multiple spin echo sequences to generate T_2_ maps. T_2_ maps were generated using a multi-slice multi-echo sequence (TR/TE = 4 s/ 6 ~ 75.4 ms, 12 echoes, matrix size = 256 x 128, FOV = 25.6 x 25.6 mm) with fat suppression (gaussian pulse, pulse length = 1.3 ms, bandwidth = 2100.5 Hz) at 14T. We utilized: $SI=Ae^{\left(-\frac{TE}{T2}\right)}$ to fit the T_2_ values to generate quantitative maps, where SI is the signal intensity and A is the amplitude when TE = 0. T_2_ weighted images were used to not only visually inspect the muscles for apparent signs of necrosis and damage, but also to qualitatively and quantitatively measure comparable regions of interest to detect changes between muscles and time points. **Magnetization Transfer (MT)** suppression ratios, or MT ratios (MTRs), were measured using the following ratio: (SI_0_ –SI_s_)/SI_0_, where SI_0_ represents the tissue signal intensity with no saturation pulse applied while SIs includes the saturation pulse. We utilized a gradient echo sequence (TR/TE = 939/2 ms, flip angle = 30°, matrix size = 256 x 256, FOV = 25.6 x 25.6 mm) with an off-resonance frequency of 5000 Hz and a saturation pulse of block pulse shape, 50 ms width, and 10 μT amplitude. For the MTI, we also suppressed the fat signal with a gaussian pulse (pulse length = 1.6 ms, bandwidth = 1750 Hz). Diffusion tensor imaging—Echo planar imaging **(DTI-EPI)** measurement (pulse duration = 2.5 ms and diffusion time = 10.4 ms) was performed to acquire series of 41 slices using following parameters: TR/TE = 500 /17.4 ms; NA = 1; FOV = 25.6 x 11.03 x 20 mm; matrix size = 128 x 64 x 41 with 6 diffusion directions. Diffusion weighted measurements were acquired with 1 b value (1000 s/mm^2^).

### MR data analysis

Image analysis of MR images was conducted using ImageJ software (http://rsbweb.nih.gov/ij), developed by the National Institutes of Health, to measure mean values of tibialis anterior (TA), gastrocnemius (GA), and soleus (SOL) muscles. Maximum cross sectional area (CSA_max_) of individual muscles was outlined to determine CSA_max_, which was calculated as the mean of the consecutive three slices having the greatest CSA for all the muscles. Furthermore, T_1_, T_2_ and MTR were calculated using the same region of interests [23]. Finally, for each muscle, four parameters i.e. three eigenvalues (λ_1_ > λ_2_ > λ_3_) and Fractional Anisotropy (FA) were calculated. Mean diffusivity (MD) was calculated by averaging the three eigenvalues and Radial diffusivity was calculated by averaging λ_2_ and λ_3_. FA is a function of all three eigenvalues that varies from 0 to 1 [26]. To improve coverage and reliability, muscles were measured for three consecutive slices at the mid-belly of the hind limb muscles [23].

### Histological analysis

Histology was conducted to correlate MRI results between various age groups of *mdx*^*4cv*^ mice. Right hind limbs were collected and fixed in 4% paraformaldehyde (PFA) solution for 24 hours while the individual muscles (TA, GA, and SOL) of the left leg were harvested and immediately frozen in optimum cutting temperature medium (OCT). The right hind limbs were subsequently decalcified in 5% formic acid for another 24 hours before rinsing and being placed into sucrose solutions (10, 20, and 30%) overnight. These right hind limbs were then frozen in OCT before being sectioned (alongside the individual muscles of the left leg) into serial, 8-μm thick sections cut with a cryostat (CM1950, Leica Biosystems Inc., Buffalo Grove, Illinois) and stained with hematoxylin and eosin (H&E) and Masson’s trichrome. All sections were examined using an 80i upright microscope (Nikon, Melville, New York). Muscle fiber cross sectional area was measured using NIH ImageJ software. All the individual muscle fibers were manually traced and fiber area was recorded for 150–200 muscle fibers in each mouse.

### Statistical analysis

All statistical analysis was conducted using Graph Pad Prism 6.0 software (GraphPad Software, USA). Values of TA, GA, and SOL muscles were compared at each time point between the right and left legs of the *mdx*^*4cv*^ and control mice. All Statistical analyses were performed using GraphPad Prism 6 software (GraphPad Software, La Jolla, CA, USA) and included one-way analysis of variance (ANOVA) followed by Tukey’s multiple comparisons test. Independent sample t test was used to make comparisons between *mdx*^*4cv*^ and control mice at 11-week time point. All data was presented in means and standard deviations with a statistical significance of p < 0.05 being accepted.

## Results

### Temporal changes in muscle cross sectional area in *mdx*^*4cv*^ and control groups

**Fig 1:**
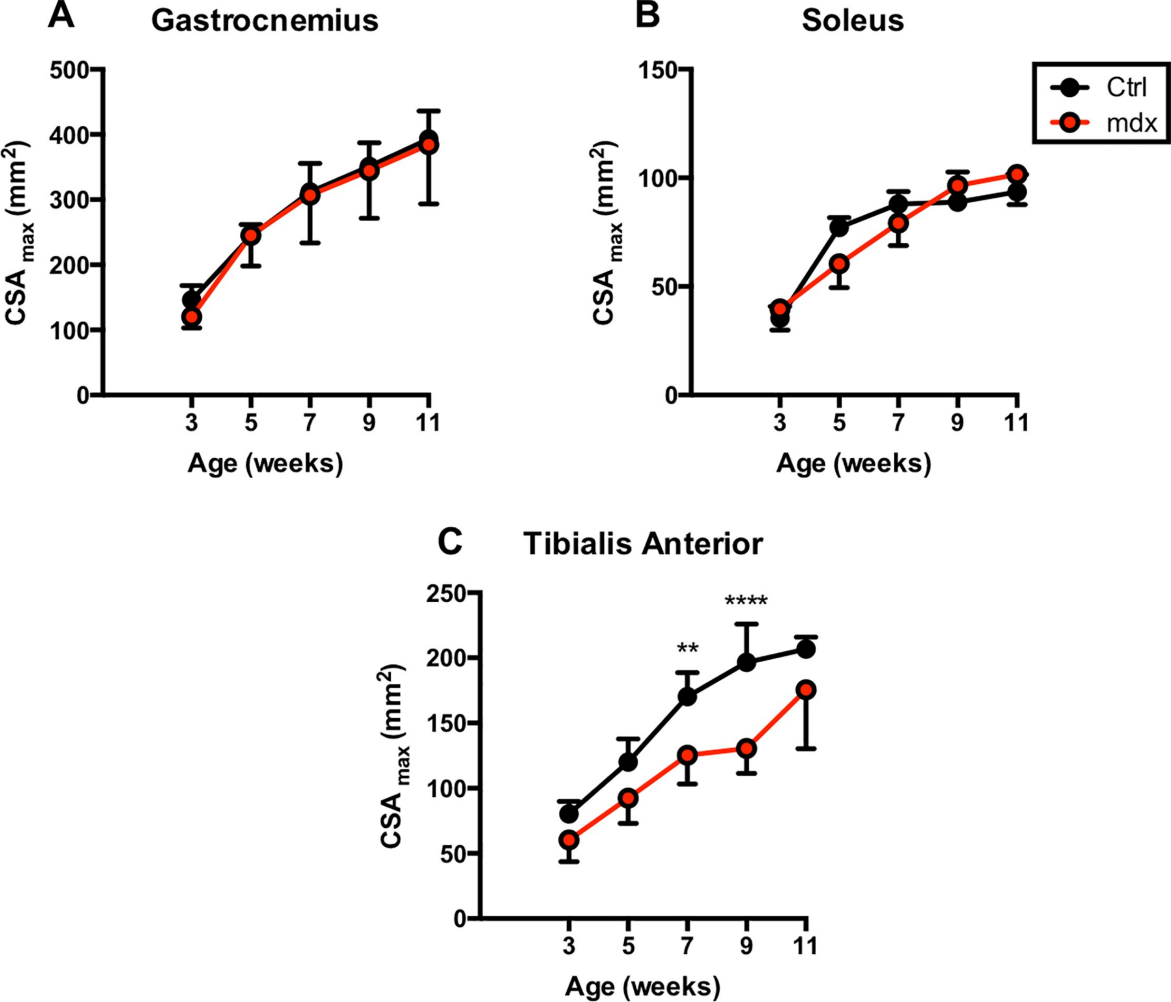
Cross-sectional area for GA, SOL, and TA muscles. Comparisons of muscle maximum cross-sectional area (CSA_max_) between *mdx*^*4cv*^ and control groups for GA, SOL, and TA muscles at each time point.

We used MRI to quantify *in vivo* differences in muscle size of dystrophic and TA, GA and Sol muscles at 3, 5, 7 and 11 weeks of age. Both *mdx*^*4cv*^ and control (ctrl) mice demonstrated age related increase in muscle size (Fig 1). Posterior compartment muscles (GA, SOL) did not show any difference between *mdx*^*4cv*^ and ctrl mice, whereas maximum cross-sectional area (CSA_max_) of TA was significantly higher in ctrl mice at 7 (ctrl vs *mdx*^*4cv*^; 170.30 ± 18.31 mm^2^ vs 125.37 ± 22.11 mm^2^) and 9 (ctrl vs *mdx*^*4cv*^; 196.50 ± 29.47 mm^2^ vs 130.60 ± 19.17 mm^2^) weeks of age.

### T_1_ and T_2_ relaxation time and MTR differences between *mdx*^*4cv*^ and control groups

**Fig 2:**
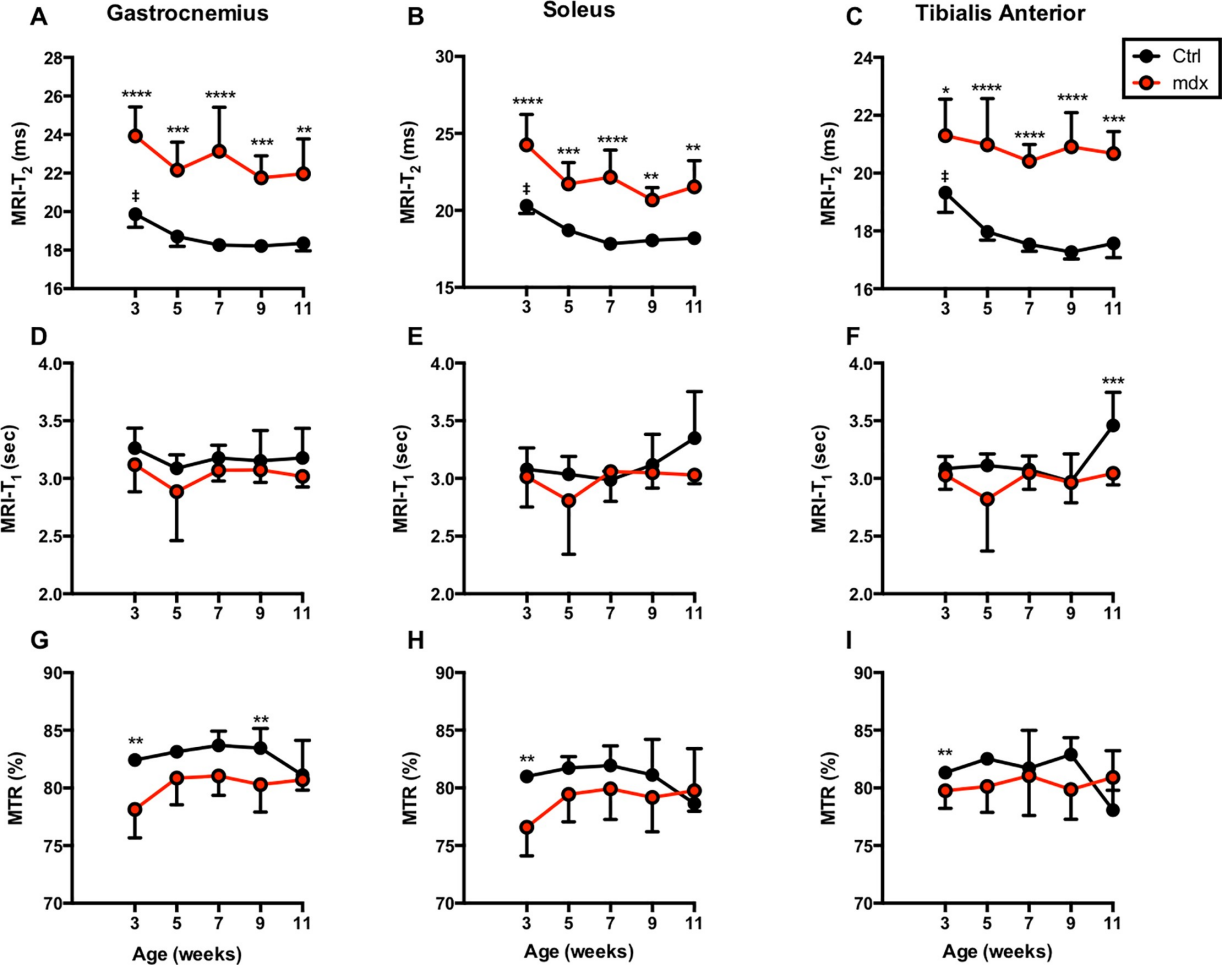
T_2_, T_1_, and MTR values analyzed for TA, GA, and SOL muscles. Graphs displaying the average longitudinal values of the *mdx*^*4cv*^ and control mice muscles in the T_2_, T_1_, and MTR measures. T_2_ values for the *mdx*^*4cv*^ mice were significantly higher at all time points versus age-matched controls. *p ≤ 0.05, **p ≤ 0.01, ***p ≤ 0.001, and ****p ≤ 0.0001. Significant higher T_2_ values were detected in ctrl mice at 3 weeks of age compared to other time-points. ‡p ≤ 0.05.

**Fig 3:**
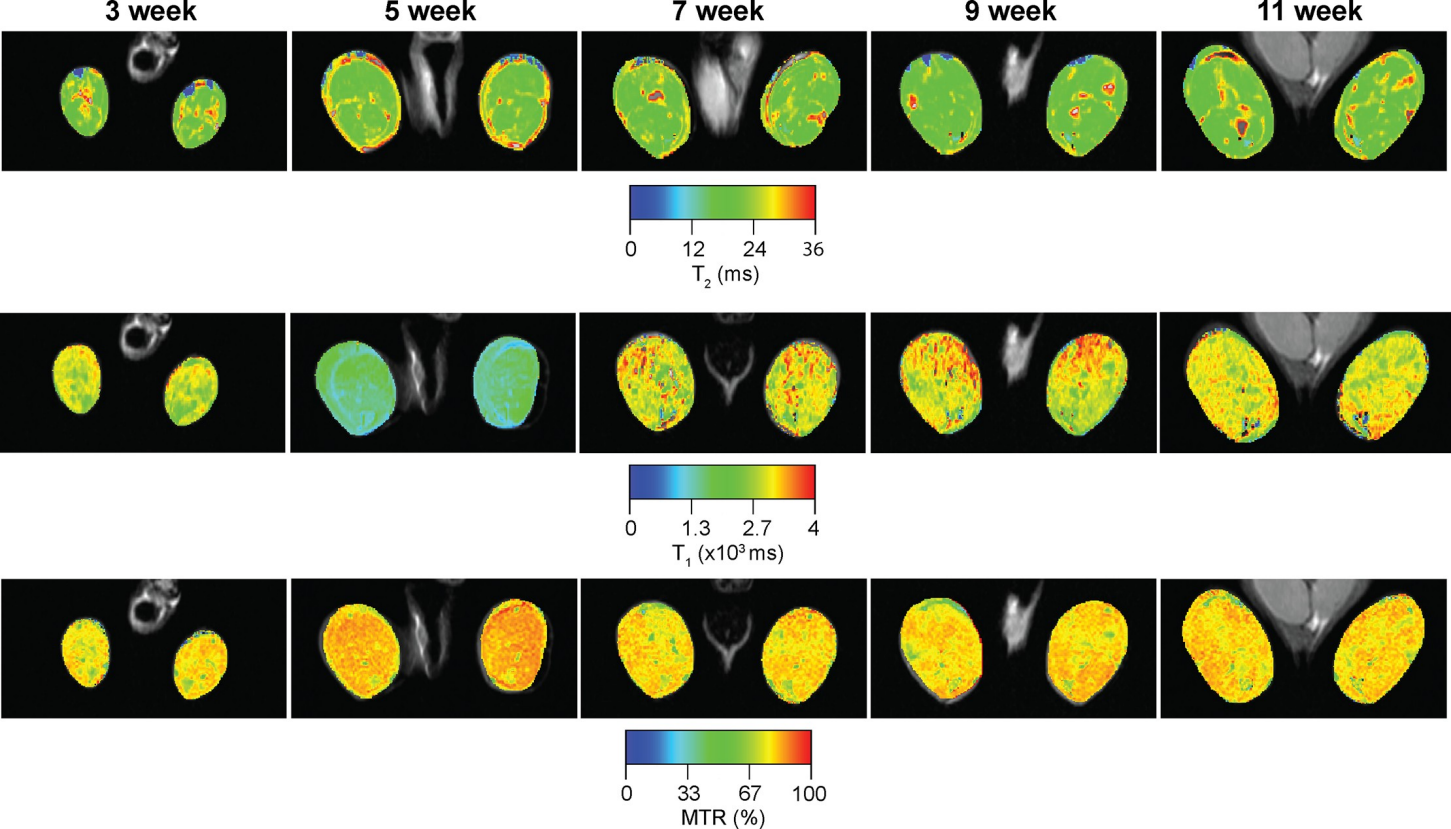
Representative T_2_, T_1_, and MTR color maps acquired from lower hind limbs of a *mdx*^*4cv*^ mouse followed longitudinally over 11 weeks. The T_2_, T_1_ and MTR maps are overlaid onto T_2_ weighted images to display the change in values for each time point.

Mp-MRI displayed significant differences when comparing different parameters between *mdx*^*4cv*^ and normal mice. Fig 2 displays the longitudinal tracking of average T_2_, T_1_, and MTR values for *mdx*^*4cv*^ mice versus controls. There were significant differences between the groups when analyzing the results of the T_2_. P-values ranged from <0.0001 to 0.0060, <0.0001 to 0.0271, and <0.0001 to 0.0097 for the GA, TA, and SOL, respectively. T_2_ measurements in control mice displayed an average decrease in relaxation time of approximately 10% between the first (3 weeks of age) and final (11 weeks of age) time points across all three muscles (TA, GA, and SOL). In *mdx*^*4cv*^ mice, the average percentage change for the analyzed muscle types was far more variable (e.g., 2.3% decrease in T_2_ of TA muscle, 4.8% decrease for GA and 9.9% decrease for SOL muscles). Statistical analyses for each group of mice from week to week revealed no significant changes in either *mdx*^*4cv*^ mice. Significant drop in T_2_ was detected in control muscles from 3 weeks (mean ± SD, TA; 19.33 ± 0.68 ms, GA;19.87 ± 0.69; SOL, 20.30 ± 0.50 ms) to 11 weeks of age (TA; 17.57 ± 0.50 ms, GA; 18.35 ± 0.39 ms; SOL, 18.19 ± 0.34 ms). T_1_ measurements revealed no significant differences between groups with control mice having slightly higher average values than the *mdx*^*4cv*^ mice with few exceptions. MTR measurements show little significance between groups, with only the earliest time points demonstrating significant differences in MTR as shown in Fig 2G, 2H and 2I. There were no significant changes within groups over the course of the study. Longitudinal quantitative T_2_, T_1_, and MTR maps overlaid on the corresponding images are displayed in Fig 3.

### Diffusion changes in *mdx*^*4cv*^ and control muscles with age

**Fig 4:**
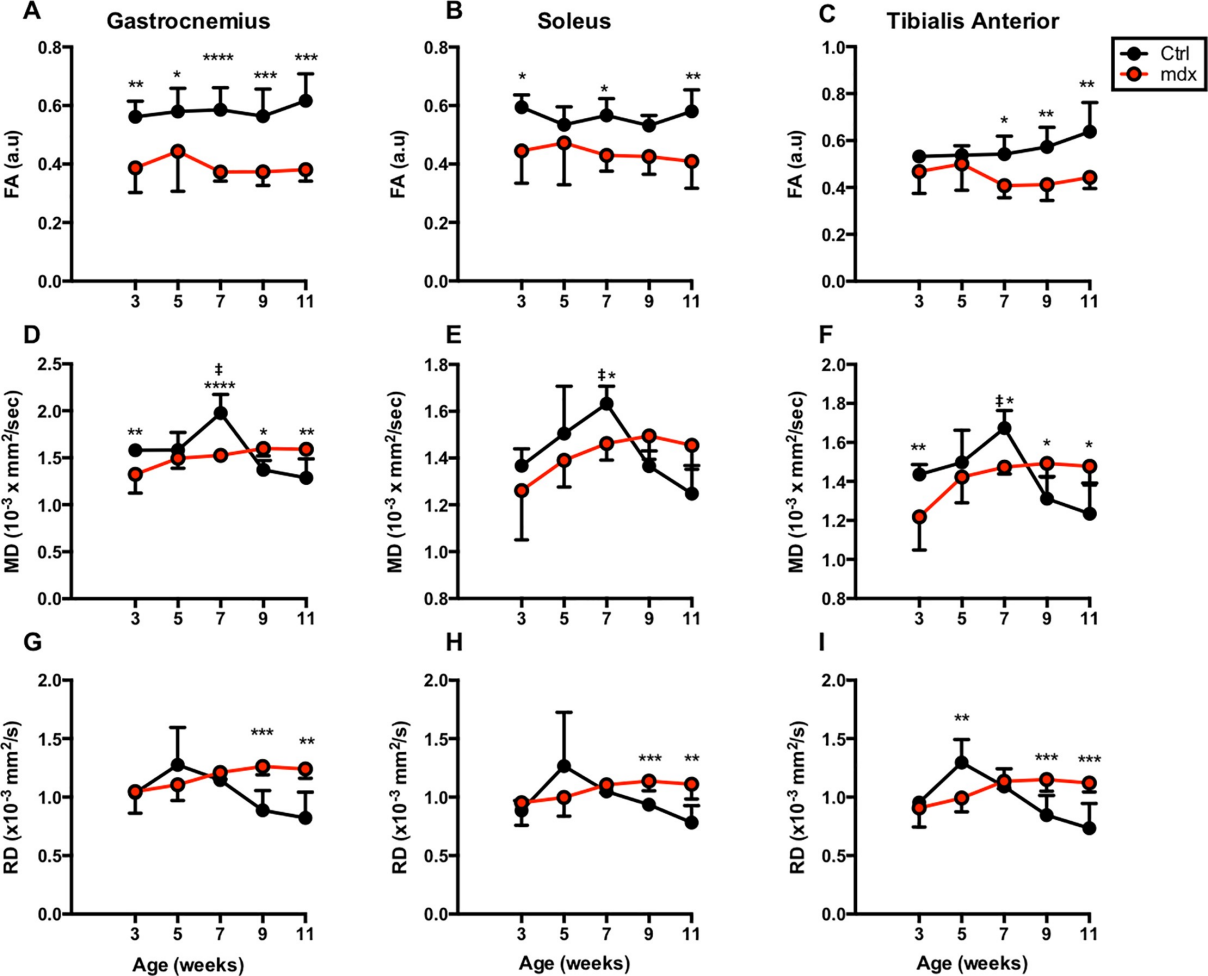
Fractional Anisotropy (FA), Mean Diffusivity (MD) and Radial Diffusivity (RD) of TA, GA, and SOL muscles. Graphs displaying average longitudinal FA, MD, and RD values for *mdx*^*4cv*^ and control muscles. FA values from GA muscles of *mdx*^*4cv*^ mice are significantly lower at all time points versus age-matched controls. *P ≤ 0.05, **P ≤ 0.01, ***P ≤ 0.001, and ****P ≤ 0.0001.

Fractional anisotropy (FA) demonstrated significant differences between *mdx* and control muscles (Fig 4A, 4B and 4C). (p-values for the GA range from <0.0001 to 0.0178, TA range from 0.0016 to 0.0196, and SOL from 0.0048 to 0.0380). FA for normal mice displayed higher and more unidirectional values compared to the *mdx*^*4cv*^ mice. This is in contrast to T_2_ measurements, which displayed cyclical behavior only for *mdx*^*4cv*^ mice while normal mice steadily dropped to stable levels. Mean Diffusivity (MD) was significantly different between ctrl and *md*x^4cv^ muscles at 3 weeks of age (ctrl vs *mdx*^*4cv*^; GA (1.58 x10^-3^ ± 0.04 x10^-3^ vs 1.32 x10^-3^ ± 0.20 x10^-3^ mm^2^/sec; p < 0.01), TA (1.44 x10^-3^ ± 0.05 x10^-3^ vs 1.22 x10^-3^ ± 0.17 x10^-3^ mm^2^/sec; p < 0.01). At 7-week time point, all three muscles of ctrl mice demonstrated significant higher ADC compared to age matched *md*x^4cv^ mice. Additionally, ctrl muscles at 7-week time point (GA; 1.98 x10^-3^ ± 0.20 x10^-3^ mm^2^/sec, SOL; 1.63 x10^-3^ ± 0.07 x10^-3^ mm^2^/sec, TA; 1.67 x10^-3^ ± 0.07 x10^-3^ mm^2^/sec) demonstrated significant higher MD compared to 3-week time point (GA; 1.58 x10^-3^ ± 0.04 x10^-3^ mm^2^/sec, SOL; 1.37 x10^-3^ ± 0.07 x10^-3^ mm^2^/sec, TA; 1.44 x10^-3^ ± 0.05 x10^-3^ mm^2^/sec) (Fig 4A, 4B and 4C). Though *mdx*^*4cv*^ mice displayed the lower MD values at 3 weeks of age, these values steadily increased by 11 weeks of age. All three muscles of ctrl mice displayed a rising trend in MD values for the first 3 time points with a decline starting at 9 weeks of age (Fig 4D, 4E and 4F). Radial diffusivity is calculated by averaging λ_2_ and λ_3_ (Fig 4G, 4H and 4I) and there were significant differences between groups seen in later time points in all three muscle types (GA with p-values of 0.0009 and 0.0015, TA with 0.0044, 0.0003, and 0.0008, and SOL with 0.0008 and 0.0082). All diffusion measurements, aside from the fractional anisotropy, share an increasing trend for *mdx*^*4cv*^ mice.

### Temporal changes in eigenvalues between *mdx*^*4cv*^ and control mice

**Fig 5:**
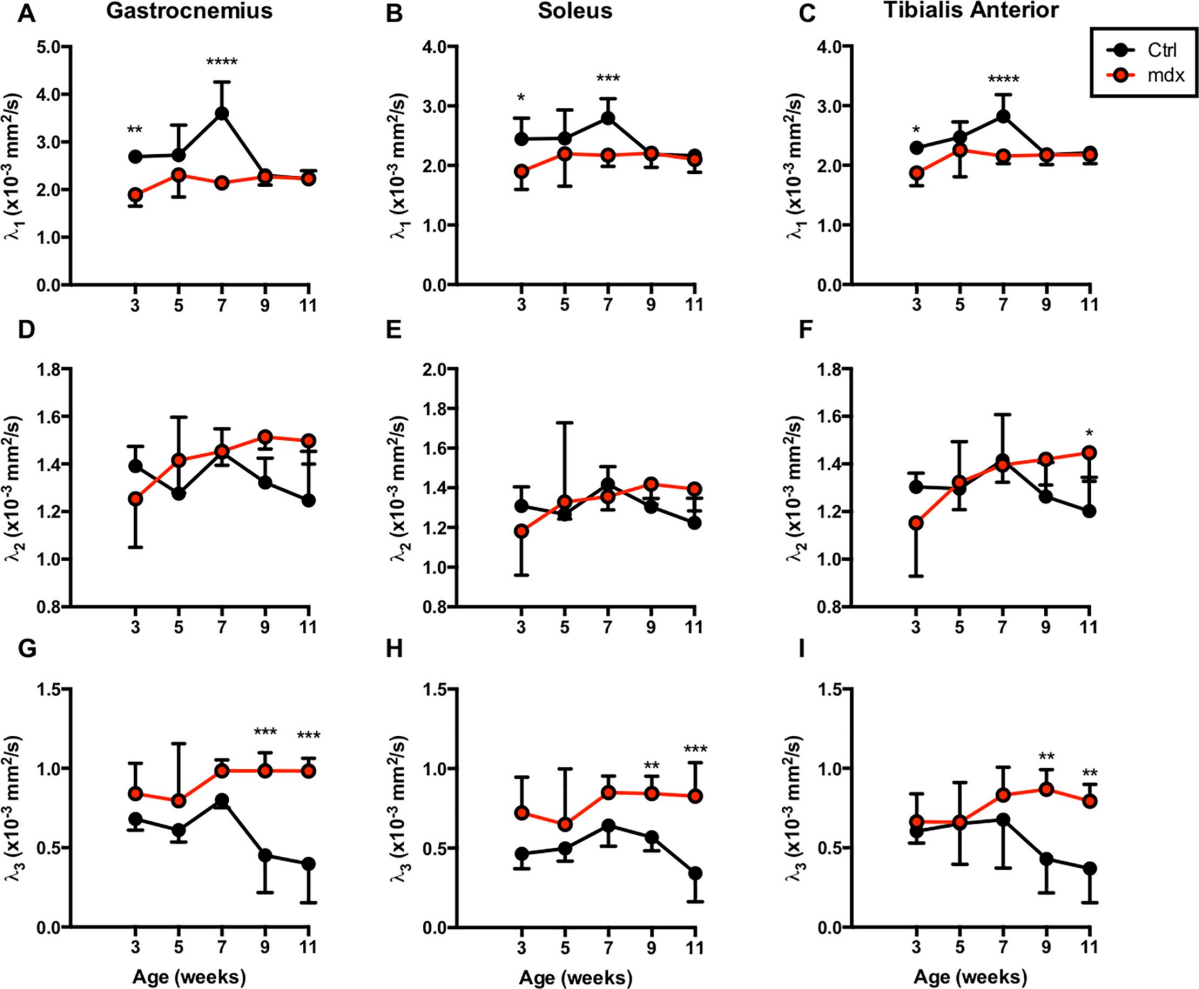
Eigenvalues analyzed for TA, GA, and SOL muscles. Graphs displaying average longitudinal eigenvalues λ_1_, λ_2_, and λ_3_ of *mdx*^*4cv*^ and control mouse muscles. λ_1_ values for *mdx*^*4cv*^ mice were significantly lower at the 3- and 5-week time points versus age-matched controls in all muscles (top panels), while λ_3_ values for *mdx*^*4cv*^ mice were significantly higher at 9 and 11 weeks (bottom panels). λ_2_ values were not significantly different between groups until the 11-week time point in the TA muscle (middle panels). *P ≤ 0.05, **P ≤ 0.01, ***P ≤ 0.001, and ****P ≤ 0.0001.

Significant difference in the primary eigenvalue (λ_1_), of ctrl and *mdx*^*4cv*^ muscles was detected at 3-week (ctrl vs *mdx*^*4cv*^, GA; 2.69 x10^-3^ ± 0.13 x10^-3^ mm^2^/sec vs 1.89x10^-3^ ± 0.24 x10^-3^ mm^2^/sec; p < 0.01, SOL; 2.45 x10^-3^ ± 0.35 x10^-3^ mm^2^/sec vs 1.90 x10^-3^ ± 0.31 x10^-3^ mm^2^/sec; p < 0.05, TA; 2.30 x10^-3^ ± 0.04 x10^-3^ mm^2^/sec vs 1.87 x10^-3^ ± 0.21 x10^-3^ mm^2^/sec; p < 0.05) and 7-week (ctrl vs *mdx*^*4cv*^, GA; 3.60 x10^-3^ ± 0.66 x10^-3^ mm^2^/sec vs 2.14 x10^-3^ ± 0.09 x10^-3^ mm^2^/sec; p < 0.0001, SOL; 2.80 x10^-3^ ± 0.33 x10^-3^ mm^2^/sec vs 2.17 x10^-3^ ± 0.19 x10^-3^ mm^2^/sec; p < 0.001, TA; 2.82 x10^-3^ ± 0.37 x10^-3^ mm^2^/sec vs 2.16 x10^-3^ ± 0.13 x10^-3^ mm^2^/sec; p < 0.0001) of age (Fig 5). No significant differences were detected in secondary eigenvalue (λ_2_) except in TA at 11-week time point (1.20 x10^-3^ ± 0.14 x10^-3^ mm^2^/sec vs 1.45 x10^-3^ ± 0.12 x10^-3^ mm^2^/sec; p < 0.05). Finally, significant differences in tertiary (λ_3_) eigenvalue were detected at 9-week (ctrl vs *mdx*^*4cv*^, GA; 0.45 x10^-3^ ± 0.24 x10^-3^ mm^2^/sec vs 0.98 x10^-3^ ± 0.11 x10^-3^ mm^2^/sec; p < 0.001, SOL; 0.57 x10^-3^ ± 0.09 x10^-3^ mm^2^/sec vs 0.84 x10^-3^ ± 0.11 x10^-3^ mm^2^/sec; p < 0.01, TA; 0.43 x10^-3^ ± 0.21 x10^-3^ mm^2^/sec vs 0.87 x10^-3^ ± 0.12 x10^-3^ mm^2^/sec; p < 0.01) and 11-week (ctrl vs *mdx*^*4cv*^, GA; 0.40 x10^-3^ ± 0.24 x10^-3^ mm^2^/sec vs 0.98 x10^-3^ ± 0.08 x10^-3^ mm^2^/sec; p < 0.001, SOL; 0.34 x10^-3^ ± 0.18 x10^-3^ mm^2^/sec vs 0.83 x10^-3^ ± 0.21 x10^-3^ mm^2^/sec; p < 0.001, TA; 0.37 x10^-3^ ± 0.21 x10^-3^ mm^2^/sec vs 0.79 x10^-3^ ± 0.11 x10^-3^ mm^2^/sec; p < 0.01) time point. Comparing eigenvalues between the two groups demonstrated significant difference in λ_1_ at 3 and 7 weeks of age and λ_3_ at 9 and 11 weeks of age.

### Histological differences between *mdx*^*4cv*^ and control mice

**Fig 6:**
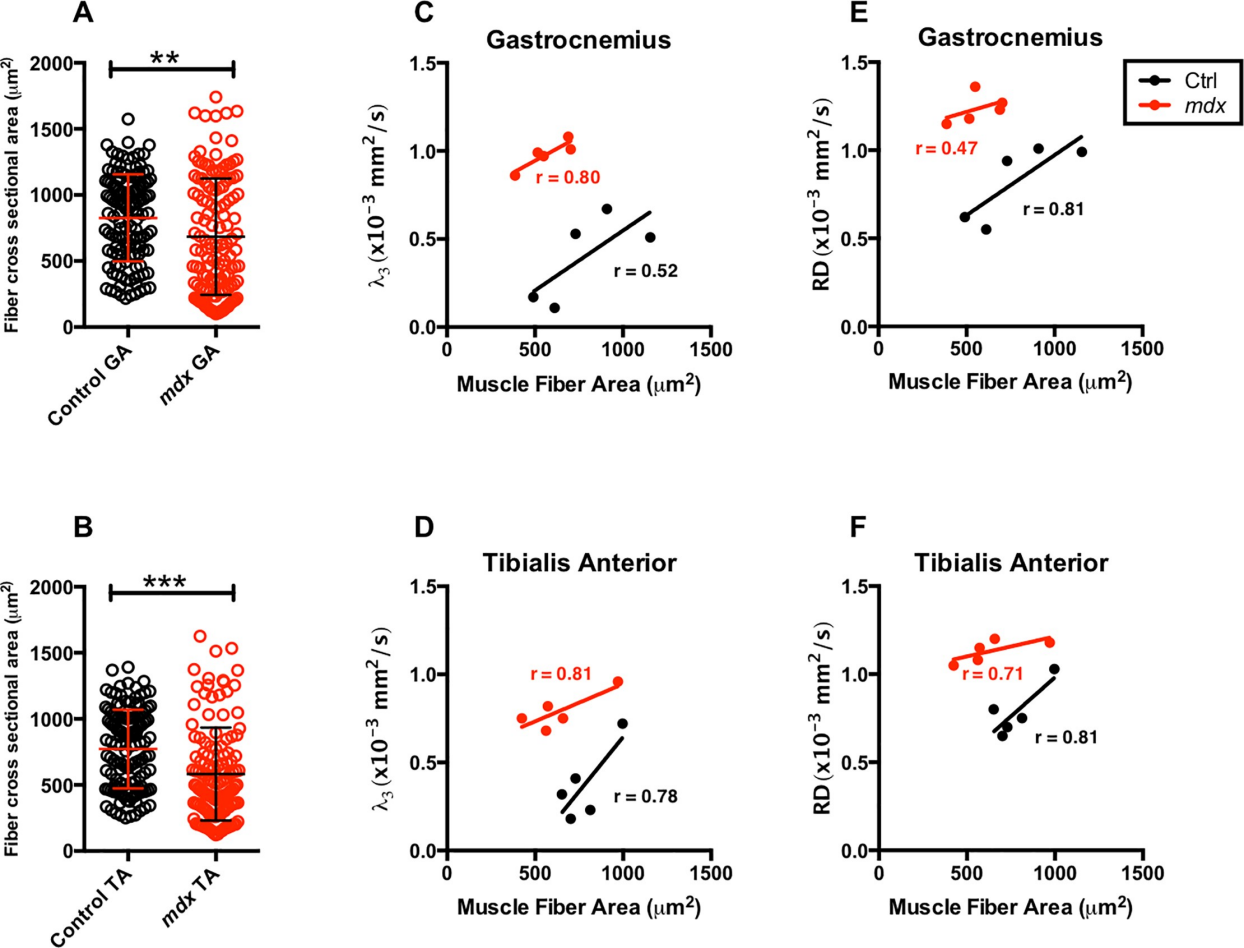
Average muscle fiber area, and λ_3_ versus average muscle fiber area for TA and GA muscles. Individual muscle fiber areas were measured for the TA and GA muscles and then averaged for comparison between groups. *Mdx*^*4cv*^ mice exhibit significantly reduced average individual muscle fiber area for both TA and GA muscles. This data was then plotted against the λ_3_ values for correlation of diffusivity across single muscle fibers and average fiber size. *P ≤ 0.05, **P ≤ 0.01, ***P ≤ 0.001, and ****P ≤ 0.0001.

**Fig 7:**
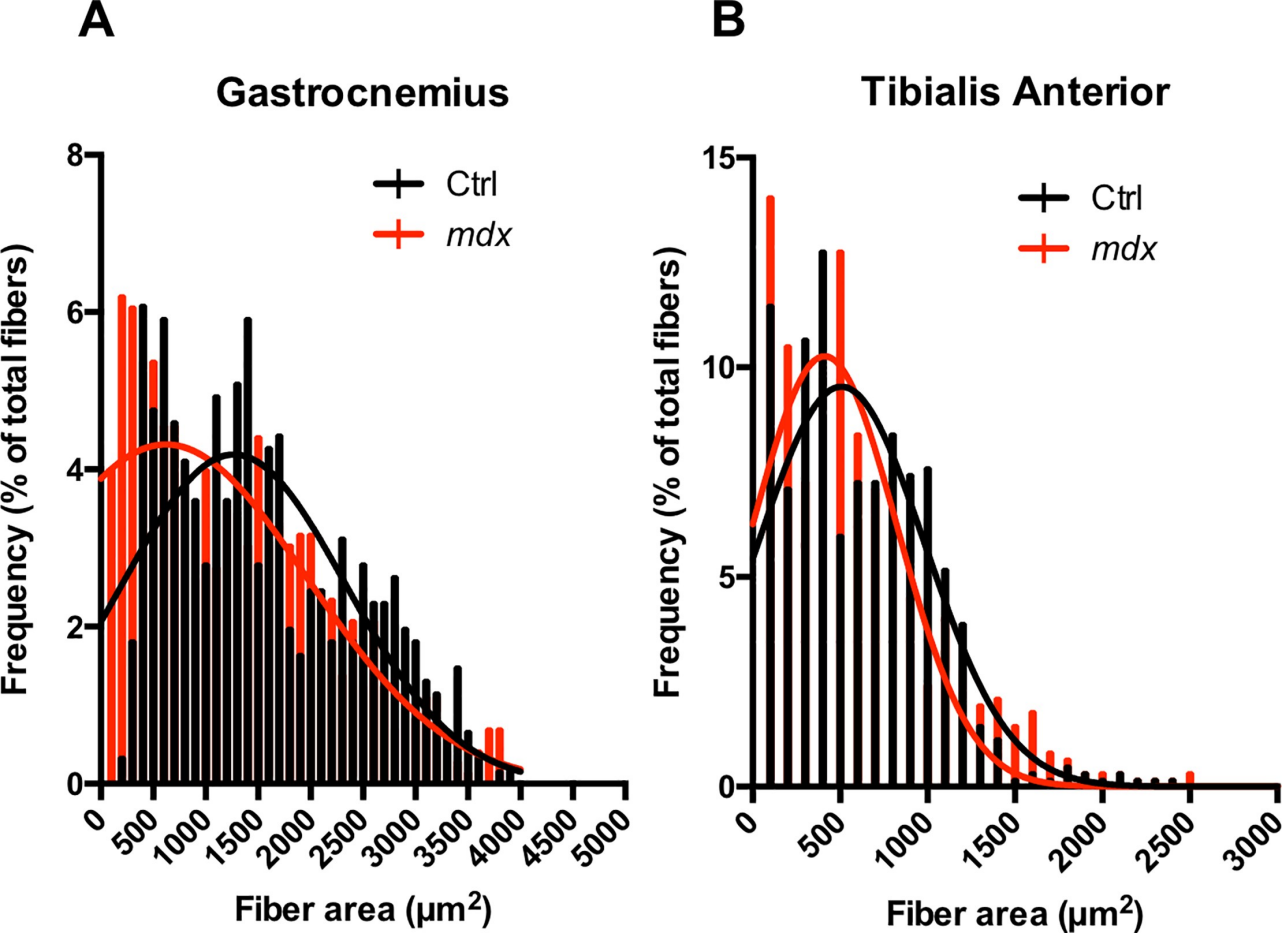
Histogram Analysis for individual muscle fiber area of TA and GA muscle fibers in 11-week old *mdx*^*4cv*^ and control mice. Muscle fiber areas measured for the TA and GA muscle differences between groups. Area values were binned into intervals of 500 μm^2^ and overlaid for both frequency and distribution comparisons.

**Fig 8:**
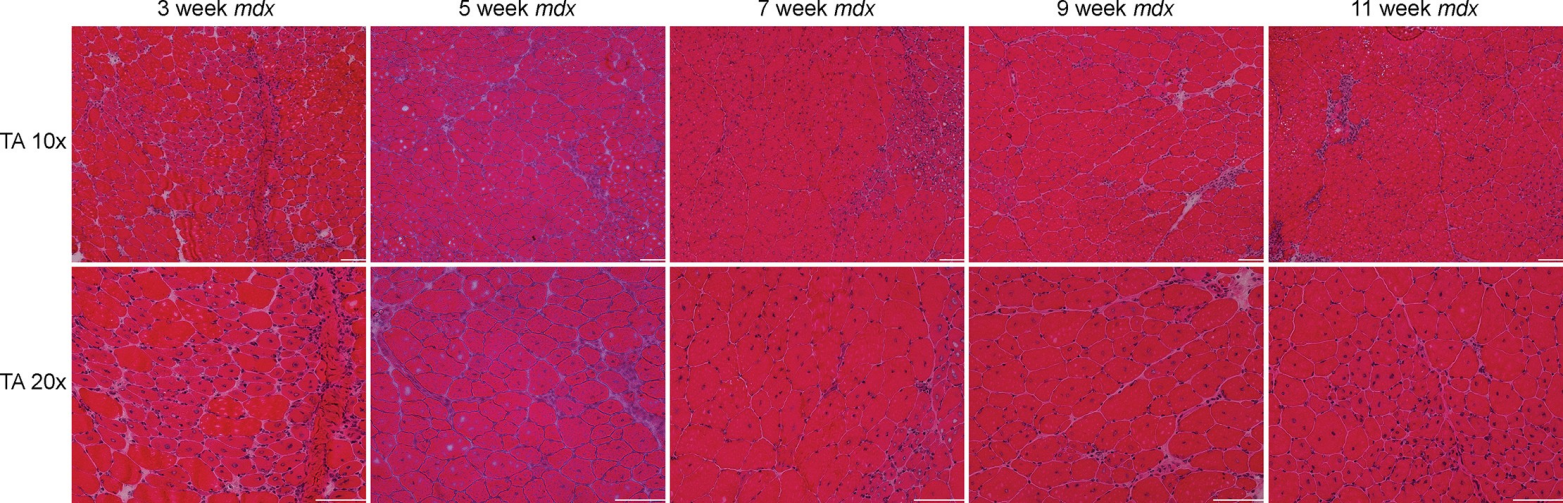
Longitudinal tissue analysis for *mdx*^*4cv*^ mice at each timing point (3, 5, 7, 9, and 11 weeks of age) at 10x and 20x magnification. Representative H&E histology images of the TA muscle in *mdx*^*4cv*^ mice. Images are taken from the same area of muscle for both 10x and 20x images (scale bar represents 100 μm).

**Fig 9:**
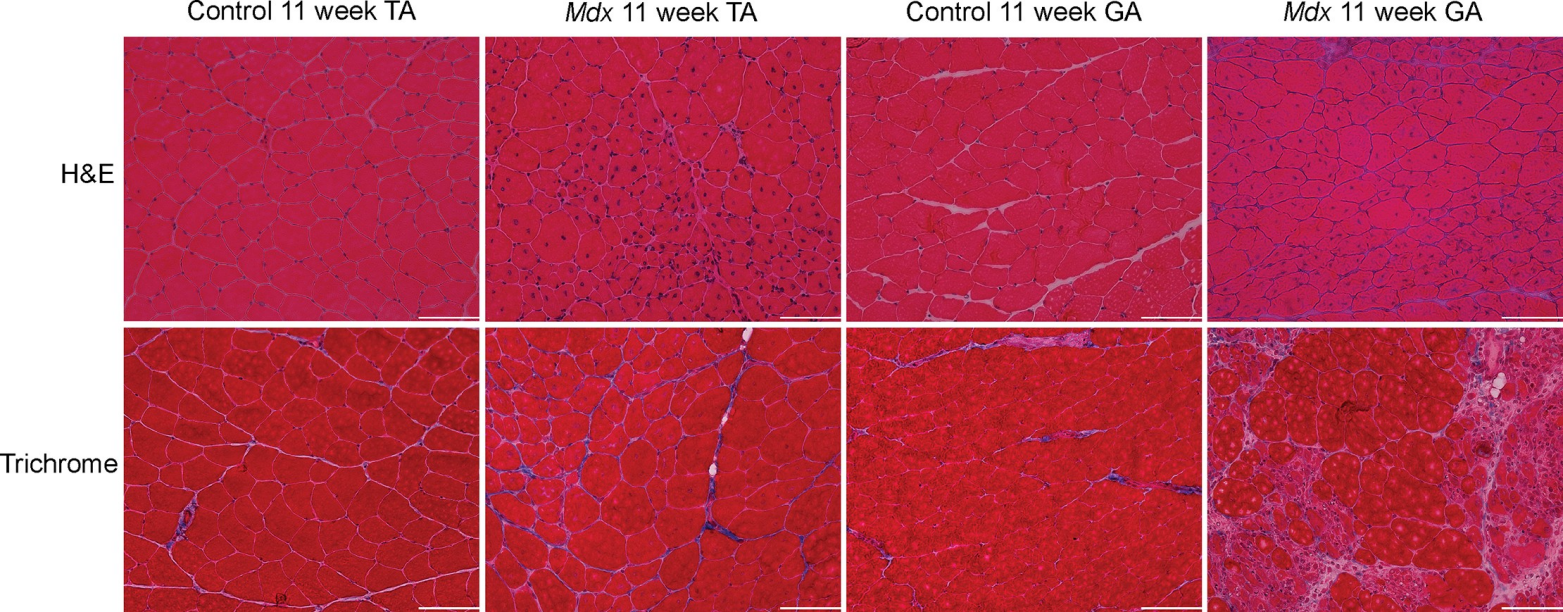
11-week comparison of TA and GA muscles of control and *mdx*^*4cv*^ mice (both H&E and trichrome staining) at 20x magnification. Comparisons of representative histology images for both groups of animals (scale bar represents 100 μm).

GA and TA muscle cryo-sections of ctrl and *mdx*^*4cv*^ at 11 weeks of age were stained with H&E stains and quantified for fiber cross sectional area. Muscle fiber cross sectional area (CSA) of *mdx*^*4cv*^ demonstrated significant smaller muscle fiber CSA compared to ctrl muscle fiber CSA at 11 week of age (ctrl vs *mdx*^*4cv*^; GA, 826.4 ± 328.6 μm^2^ vs 684.2 ± 440.6 μm^2^, p < 0.01; TA, 771.7 ± 298.0 μm^2^ vs 582.2 ± 351.3 μm^2^, p < 0.01; Fig 6A and 6B). Additionally, there were positive correlation between λ_3_ and muscle fiber CSA of ctrl and *mdx*^*4cv*^ GA (r = 0.52 and r = 0.80) and TA (r = 0.78 and r = 0.81) (Fig 6C and 6D). Furthermore, a positive correlation was detected between RD and muscle fiber CSA of ctrl and *mdx*^*4cv*^ GA (r = 0.81 and r = 0.47) and TA (r = 0.81 and r = 0.71). Quantitative analysis of muscle fiber CSA, frequency distribution, of GA and TA muscle fibers displayed a leftward shift (Fig 7A and 7B). Finally, qualitative inspection of muscle fibers showed higher number of centrally nucleated fibers in *mdx*^*4cv*^ than ctrl muscles (Fig 8). Masson’s trichrome staining can reveal fibrous connective tissue and collagen (stained as blue)–however, there is little fibrosis to be expected in mice of this age (Fig 9).

## Discussion

Utilization of mp-MRI has been shown to be a valuable tool in the investigation of skeletal muscle pathology and the present study used mp-MRI to explore young adult *mdx*^*4cv*^ mouse muscle pathology versus healthy age-matched controls. Previous studies have demonstrated that the cyclical changes occur in skeletal muscles of young dystrophic mice [17, 27]. However, these studies did not begin as early as 3 weeks of age nor used a multi-parametric characterization approach. The results of the present study demonstrate that 1) T_2_ continues to be the most sensitive parameter for observing dramatic changes in dystrophic muscle tissue 2) FA is particularly sensitive during this early phase and 3) radial diffusivity and eigenvalue comparisons (λ_1_ and λ_3_ in particular) display moderate sensitivity for detecting dystrophic muscle changes.

### Temporal behavior of MR parameters

T_2_ measurements of TA, GA, and SOL muscles showed significant differences when comparing *mdx*^*4cv*^ mice compared to age-matched controls. This confirms previous findings demonstrating increased sensitivity of T_2_ measurements in both preclinical and clinical models of DMD [23, 24, 28]. There was an age dependent T_2_ decline in healthy control mice from 3 weeks to 5 weeks of age after which the T_2_ values were stable. However, *mdx*^*4cv*^ mice underwent demonstrated cyclical changes in the lower hind limb muscles. In fact, increased body of evidence has reported that younger *mdx* mice go through cycles of inflammation, necrosis, and regeneration between 3–10 weeks of age, with decreased but ongoing cycles of necrosis and regeneration thereafter [8, 29]. Additionally, it has been reported that there is 2-4-fold increase in *utrophin* protein in dystrophic muscles and it localizes at the sarcolemma of regenerating fibers [30, 31]. The T_2_ values from *mdx*^*4cv*^ mice were always elevated when compared to control mice–a pattern which was not reflected as strongly in other MR parameters. The average T_2_ values in our study ranged between 22–26 ms in *mdx* mice and 17–20 ms in control muscles were smaller as compared to previously published (~ 30 ms in *mdx* mice and 27 ms in controls measured at 4.7T) [24]. Tissue T_2_ has been shown to decrease with increase in magnetic field strength thus providing one explanation of difference between the two studies. The T_2_ relaxation in skeletal muscle has been attributed to three primary signal components (<5, 25–45, and >100 ms) with the intermediate value range contributing most to the overall signal [32–35]. In particular, these intermediate values are related to the hydration of macromolecules as well as the presence of intracellular- and extracellular water. Utilizing T_2_ scans to effectively identify responses for such intracellular and extracellular water in conjunction with fat suppression has been hypothesized to reflect either increase of extracellular compartments, necrotic regions, or inflammation and edema [36–39], but not from fatty infiltration [40–42]. As seen in the values obtained for normal muscle, T_2_ values were at their highest in the earliest weeks of the study and gradually decreased towards later time points. These values stabilized and previous research indicates that such T_2_ values are associated with normal hydration of the extracellular space of skeletal muscle [33, 34]. Because the T_2_ can be readily affected by any of such changes, the utilization of mp-MRI to capture a more nuanced understanding of possible biomarkers is highly informative.

Magnetization transfer ratio (MTR) is a measure of the efficiency of magnetization transfer between bound (“restricted”) and adjacent mobile (“free”) water protons. When tissue is damaged, there are fewer hydrogen atoms bound to macromolecules, which leads to a decreased magnetization transfer [43]. Additionally, because muscle fibers that are well organized can be expected to have an increased abundance of macromolecules, MTR should be higher in the muscles of healthy controls [44]. Furthermore, studies using MT imaging have suggested its utility in evaluating skeletal muscles [45, 46]. While not significantly higher at all time points, the MTR measurements displayed a general trend with lower values found in the *mdx*^*4cv*^ group versus controls at earlier time points. MT could still prove to be useful in measurements of *mdx* mice because fatty tissue does not show MT due to the lack of water molecules [44]. Additionally, MT has been shown to be sensitive to fibrosis formation in other diseases such as Crohn’s disease [47] and pancreatic tumors [48, 49]. Thus, measurements of MT in *mdx*^*4cv*^ mice at a young age could be further refined to capture early fibrotic tissue formation in young muscle as well as fibrosis seen in older *mdx*^*4cv*^ mice for useful translation to human studies of DMD where fatty infiltration and higher levels of fibrosis also occur in skeletal muscle.

Increased body of evidence suggests that any insult to skeletal muscles may lead to alteration in FA and corresponding changes in diffusivity measures [50–52]. Although, techniques like T_2_ and MT imaging are sensitive to various underlying pathological processes, they are not ideal for quantifying changes in muscle fiber morphology [52]. Specifically, eigenvalues have been suggested as indicators of water diffusion across various axes of muscle fibers [53], with λ_1_ representing diffusion along the long axis of the fiber [54, 55]. Galbán et al. demonstrated that λ_2_ represents diffusion within the endomysium and λ_3_ represents diffusion within the cross section of a muscle fiber [53]. Furthermore, a study by Zhang et al. has demonstrated decrease in secondary and tertiary eigenvalues in a complete denervation and chronic denervation models [56]. In addition, Heemskerk et al., in an ischemic model, have demonstrated an increase in mean diffusivity and a correlation between swollen myocytes and smallest eigenvalue, i.e. λ_3_ [51]. Our findings are in-line with these previous observations and suggest that secondary and tertiary eigenvalues are markers of muscle fiber atrophy. The diffusion and MTR values, in conjunction with the three eigenvalues themselves suggest diffusion along the axis of individual muscle fibers is disrupted in *mdx*^*4cv*^ mice while diffusion perpendicular to individual and bundles of fibers is increasing. Possible explanations for these observations include that the presence of compromised myofiber membranes could increase diffusion out of myofibers; as well as that areas of necrotic/degenerating fibers would greatly increase multi-directional diffusion until regeneration, fibrosis or adipogenesis occurs.

Muscle fiber cross-sectional area analysis revealed a significant difference in TA and GA muscles of *mdx*^*4cv*^ and control mice. Additionally, at 11 weeks of age we observed greater degree of variability in muscle fiber size of *mdx*^4cv^ mice compared to age-matched control mice. This could be attributed to cyclical periods of degeneration and regeneration, which leads to higher number of smaller fibers and occurrence of hypertrophic fibers [8, 57]. Finally, the analysis of the fiber size distribution revealed a tendency of a shift toward a higher number of smaller myofibers in *mdx*^*4cv*^ mice compared to control mice. Our findings are in agreement with previously published results [57, 58].

The study had limitations that should be acknowledged. The multi-parametric nature of the study meant that because of the many parameters being acquired, the scans had to be modified and optimized in order to ensure the mouse’s condition did not deteriorate due to excessively prolonged anesthesia. For example, the maximum TR of 5.5 seconds used for T_1_ determination was slightly short considering the T1 of muscle is close to 3 seconds. This in turn affects the quality of the maps being used for the measurements, which may increase standard deviation. Future studies should focus on optimizing scan protocols further, particularly for T_1_ measurements with longer TR (9 seconds or longer) than 5.5 seconds and ADC measurements using recent advances in DWI. Additionally, such studies could also incorporate more nuanced histological assessment for additional corroboration. Finally, future studies could operate at preclinical/clinical field strengths for direct translation into tracking of human trials of DMD treatment.

## Conclusions

Mp-MRI can be used to identify quantifiable differences between *mdx*^*4cv*^ and normal mice that can be monitored over time noninvasively. Mp-MRI parameters such as T_2_, FA, radial diffusivity, and eigenvalues are sensitive and significantly different between *mdx*^*4cv*^ and normal groups and could prove highly useful in preclinical settings for monitoring disease progression and response to treatments. Radial diffusivity, MT, and eigenvalue analysis also show promise for understanding cellular differences between normal and dystrophic muscle. This multi-parametric data suggests that many MR techniques could be used in preclinical and clinical models of muscular dystrophy treatment.

## Supporting information

S1 File Data pages. (XLSX)
